# Variability of Clinical Practice in the Third Stage of Labour in Spain

**DOI:** 10.3390/jcm8050637

**Published:** 2019-05-09

**Authors:** Inmaculada Ortiz-Esquinas, Juan Gómez-Salgado, Ana I. Pascual-Pedreño, Julián Rodríguez-Almagro, Juan Miguel Martínez-Galiano, Antonio Hernández-Martínez

**Affiliations:** 1 Department of Obstetrics & Gynaecology, Alcázar de San Juan, 13600 Ciudad Real, Spain; inmaores@hotmail.com (I.O.-E.); ginemancha@hotmail.com (A.I.P.-P.); antomatron@gmail.com (A.H.-M.); 2 Department of Sociology, Social Work and Public Health, University of Huelva, 21071 Huelva, Spain; salgado@uhu.es; 3 Safety and Health Postgraduate Programme, Universidad Espíritu Santo, Guayaquil 091650, Ecuador; 4 Department of Nursing, Ciudad Real Nursing School. University of Castilla-La Mancha, 13071 Ciudad Real, Spain; 5 Department of Nursing, University of Jaen, 23071 Jaen, Spain; jgaliano@ujaen.es; 6 Consortium for Biomedical Research in Epidemiology and Public Health (CIBERESP), 28029 Madrid, Spain

**Keywords:** manual removal of placenta, professional practice, postpartum haemorrhage, third stage of labour, uterotonic agents, patient safety, quality improvement

## Abstract

Clinical practice guidelines recommend the active management of the third stage of labour, but it is currently unknown what practices professionals actually perform. Therefore, the aim of this study was to determine the variability of professional practices in the management of the third stage of labour and to identify any associated professional and work environment factors. A nationwide cross-sectional study was performed with 1054 obstetrics professionals between September and November 2018 in Spain. A self-designed questionnaire was administered online. The crude odds ratios (OR) and adjusted odds ratios (ORa) were estimated using binary logistic regression. The main outcome measures were included in the clinical management of the third stage of labour and they were: type of management, drugs, doses, routes of administration, and waiting times used. The results showed that 75.3% (783) of the professionals used uterotonic agents for delivery. Oxytocin was the most commonly administered drug. Professionals who attend home births were less likely to use uterotonics (ORa: 0.23; 95% confidence interval (CI): 0.12–0.47), while those who completed their training after 2007 (ORa: 1.57 (95% CI: 1.13–2.18) and worked in a hospital that attended >4000 births per year (ORa: 7.95 CI: 4.02–15.72) were more likely to use them. Statistically significant differences were also observed between midwives and gynaecologists as for the clinical management of this stage of labour (*p* < 0.005). These findings could suggest that there is clinical variability among obstetrics professionals regarding the management of delivery. Part of this variability can be attributed to professional and work environment factors.

## 1. Introduction

Postpartum haemorrhage (PPH) is the cause of 27.1% of maternal deaths worldwide and is the leading cause of maternal mortality and morbidity. This complication occurs in the third stage of labour [1,2]. Two different ways of managing the third stage of labour have been proposed: expectant, in which the placenta is expelled by the mother [3], and active, which uses uterotonic drugs either exclusively or in combination with controlled cord contraction [4,5,6].

In a consensus statement published in 2004, the International Confederation of Midwives (ICM) and the International Federation of Gynaecologists and Obstetricians (FIGO) recommended active management in the third stage of labour to prevent PPH [4]. This recommendation was included in important Clinical Practice Guidelines (CPGs) such as those drawn up by the National Institute for Health and Care Excellence (NICE) [7] and those by the World Health Organization (WHO) [8]. However, although active management is strongly recommended, not all professionals use this process systematically [9,10,11].

When active management is the chosen option, there are multiple alternatives when it comes to manoeuvres and drugs [6,12,13,14,15]. Regarding cord traction, some authors recommend the Brandt–Andrews manoeuvre [16]. However, it is not known whether this method is better than other procedures or to what extent it is used. Also, with regard to retained placenta, which can be defined as the lack of expulsion of the placenta within the first 30 min of delivery of the infant, when the third stage of labour is actively managed [7,17], there is lack of knowledge about how long to wait before a gynaecologist should assess the need for manual removal in both active and spontaneous management. Although the objective of this study is not to evaluate the effectiveness of these practices, it would be interesting to determine which practices professionals actually perform and under which circumstances these are carried out.

Knowledge in this regard could be of great interest for both professionals and healthcare institutions, as not many studies have been published which evaluate the procedures carried out during the third stage of labour [17,18] and the differences between professionals as to how these are performed [19,20]. Therefore, this study was designed with the aim of determining whether there is variability in the professional practice during the third stage of labour and identifying the professional or work environment factors that may be associated with the various alternatives involved in the management of this stage of labour.

## 2. Material and Methods

### 2.1. Design and Selection of Study Subjects

Observational cross-sectional study with obstetrics professionals (gynaecologists, midwives, and trainees in both specialities) in 2018.

Obstetrics professionals who exclusively worked in primary care and did not attend childbirths were excluded. 

To estimate the sample size, the following criteria were considered: a reference population of 16,361 individuals (9013 midwives, 5616 obstetricians and 1732 trainees in either speciality: 743 in midwifery and 989 in obstetrics) according to official statistics and the number of training places offered by the Spanish Ministry of Health, Consumption and Social Welfare [21,22]. As it was a multiple choice questionnaire in which the prevalence of each response option was unknown, a prevalence of 50% was used for being the criterion that requires the largest sample size, as well as a confidence level of 95% and a precision or absolute error of 3%, giving a minimum sample size of 1002 study subjects. For this estimation, the EPIDAT 4.1 software was used. 

### 2.2. Information Sources

For data collection, a self-designed and anonymous online questionnaire was used, containing 35 items (2 open-ended questions and 33 closed-ended questions) on sociodemographic, professional and work environment factors, and on the different ways of managing delivery.

The questionnaire had been previously piloted and was distributed to obstetrics professionals in Spain via the Federation of Midwives’ Associations of Spain (FAME) and the National Midwives’ Association. The directors of these associations were involved in publicising the project and attracting participants. The questionnaire was also distributed through several scientific societies of obstetrics. Before starting the questionnaire, the health professionals were required to read an information sheet about the study, its aims and any other relevant information, and gave their consent to participating in the study by completing and handing in the questionnaire.

After giving their consent, they were given the instructions to complete the questionnaire. An email address was offered to give answer to any questions or issues raised in relation to filling out the questionnaire.

The following variables were collected:

The main dependent variable was the type of delivery (physiological/cord traction only/administration of uterotonic drugs/combination of traction and administration of uterotonic drugs). This variable was later categorised within the variable "Use of uterotonics in the third stage of labour (No/Yes)”, as this was the most decisive element in preventing PPH during active management [13,23]. The “No” category included physiological delivery or delivery with cord traction only, while the “Yes” category included the administration of uterotonics whether alone or in combination with cord traction. The other dependent variables and their categories are shown in Tables 2, 3, Table A1 and Table A2.

The independent variables were: age, gender, profession (Midwife/Trainee midwife/Gynaecologist/Trainee gynaecologist), works at a public centre (No/Yes), works at a private centre (No/Yes), attends home births (No/Yes), works in primary care (No/Yes), number of births per year at the centre they work at (<500 births/500–1000 births/1000–2000 births/2000–4000 births/>4000 births), presence of trainee professionals at the centre they work at (No trainee professionals/Trainee midwives only/Trainee gynaecologists only/Trainees in both specialities) and year of completion of training (Before 2007/After 2007/In training). 2007 was chosen as a cut-off point as this was the year when the NICE CPGs were published [7], which are reference guidelines for obstetrics professionals and an intermediate step between the first FIGO, the ICM consensus statement in 2004 [4], and Spain’s GPC (Clinical Practice Guidelines) [24]. Also, in 2007, experiments had already been documented in Spanish centres with the inclusion of active management by following protocols [25]. 

### 2.3. Statistical Analysis Used

First, a descriptive analysis was undertaken using absolute and relative frequencies. For those questions related to the practices during the third stage of labour, and with the aim of improving the representativeness of the sample, a factor analysis by weighting the profession variable was used. The weighting factor was obtained by dividing the theoretical sample according to the total distribution of professionals by the real sample obtained in the study. Next, a bivariate analysis of the different sociodemographic and professional factors in relation to the use of pharmacological delivery was done using binary logistic regression. Then, a multivariate analysis was done through binary logistic regression using SPSS forward and backward selection, and potential confounders were included in the analysis. The crude (OR) and adjusted (ORa) odds ratios were estimated with a respective confidence interval of 95% (CI 95%). Finally, a sub-analysis was done to compare different care practices in the immediate postpartum period according to each profession (midwives/gynaecologists) and whether the professionals attended home births (No/Yes), by using the chi-square test or the Mann–Whitney U test, depending on the type of variable.

## 3. Results

In the study 1054 professionals took part, of which 75.6% (797) were midwives, 11.0% (116) were gynaecologists and 13.5% (142) were trainee midwives or trainee gynaecologists. 89.2% (940) of the sample were women, 26.7% (281) had completed their training in their speciality before 2007, 4.1% (43) attended home births, and 27.2% stated that, at their work centre, there was no established protocol for the third stage of labour. Table 1 gives a detailed description of the professional and work environment factors and the response rate of the whole population.

With regard to the practices carried out during the deliveries, 17.1% (180) practised expectant or physiological management, while 7.6% (80) used active management with controlled cord traction only, 25.3% (256) active management with uterotonics only, and 50.0% (527) used active management with both uterotonics and controlled cord traction; 54.2% (411) used the Brandt–Andrews manoeuvre during cord traction, 25.9% (273) waited 60 minutes before asking the gynaecologist to advice manual removal of the placenta in spontaneous deliveries, and 84.3% (644) of professionals that administered uterotonics during the third stage waited 30 minutes before asking a gynaecologist to advice manual removal of the placenta.

The most commonly administered drug was oxytocin in 71.6% of cases (755). Of the professionals that used uterotonics in the third stage of labour, 72.4% (557) always did so, 22.5% habitually did so (173), and 4.7% (36) only did so when there were risk factors. When applying an analysis through the weighting factor to the profession variable, no relevant differences were found as compared to the non-weighted analysis. Table 2 and Table 3 show the delivery management factors.

Next, the relationship between the use of uterotonics in the third stage of labour (No/Yes) and professional and work environment factors was analysed. In the multivariate analysis, it was observed that professionals that attended home births used uterotonic drugs for delivery less frequently (adjusted odds ratio (ORa): 0.23; 95% confidence interval (CI): 0.12–0.47) than those who did not attend home births. Conversely, trainees were more likely to use uterotonics, with an ORa of 1.94 (95% CI: 1.13–3.34), and those who completed their training after 2007 were more likely to use uterotonics than those who had completed their training before 2007, with an ORa of 1.57 (95% CI: 1.13–2.18). It was also seen that the greater the number of births at the centre the professional works at, the greater the probability of uterotonics being used. Professionals from centres with more than 4000 births per year showed an increased probability, with an ORa of 7.95 (95% CI: 4.02–15.72), those from centres with between 2000 and 4000 births per year also had an increased probability of using uterotonics, with an ORa of 4.89 (95% CI: 2.84–8.43), those who worked in centres with between 1000 and 2000 births presented an ORa of 1.98 (95% CI: 1.20–3.24), and those from centres with between 500 and 1000 births showed an ORa of 2.12 (95% CI: 1.20–3.67), as compared to those centres with less than 500 births per year. The bivariate and multivariate analyses are shown in Table 4.

Finally, a sub-analysis was conducted to determine the differences in practices in the postpartum period between professionals who attend home births and those that do not, and between midwives and gynaecologists. In the first comparison, statistically significant differences were found between all of the evaluated variables. Professionals who attended home births were less likely to use uterotonics (*p* = 0.002), less likely to use oxytocin after delivery (*p* < 0.001), used lower doses of oxytocin (*p* < 0.001) and had greater waiting times for both spontaneous delivery (*p* < 0.001) and when uterotonic drugs were used (*p* < 0.001) before initiating manual removal of the placenta, as compared to professionals who did not attend home births (Table A1).

In the second comparison, statistically significant differences were found between three of the assessed variables. Midwives were less likely to use uterotonic drugs (*p* = 0.008) and their waiting time was longer for both spontaneous deliveries (*p* < 0.001) and when uterotonic drugs were used (*p* < 0.001) before initiating manual removal of the placenta, as compared to gynaecologists (Table A2).

## 4. Discussion

### 4.1. Main Findings

In our study, 75.3% of professionals used uterotonics in deliveries, with a high variability in the type of drug, dose, route of administration and manoeuvres used. Furthermore, the use of uterotonics was associated with certain professional factors such as the time since completing the training, the number of births at the centre they worked at, and whether or not they attended home births. High variability was also observed with regard to the cord traction technique and to waiting times before asking a gynaecologist to advice the need for manual removal of the placenta.

### 4.2. Interpretation

In 2007, the NICE CPGs on care during childbirth recommended active management of the third stage of labour [7], as the Spanish CPGs on care during normal childbirth also stated in its publication of 2010 [24]. Despite these recommendations, some professionals opt for expectant management based on the fact that this method contributes to a more natural childbirth experience, the belief that active management is unnecessary in low-risk women, and the desire to avoid the effects associated with the use of the most habitual uterotonics [26]. In 2018, Schorn et al. identified that active bleeding, current recommendations or guidelines, and maternal or family preferences are the variables that influence clinical decisions on how to manage this stage [27].

In 2007 and 2009, the EUPHRATES Group published the results of a study aimed at determining practices in the management of the third stage of labour and the immediate management of postpartum bleeding [18], as well as the length of time before manual removal [17] following a vaginal birth in maternity units of 14 European countries. Spain participated in this study. However, it was only conducted in maternity units of Catalonia. The conclusions of the first publication were that the use of uterotonics in the management of the third stage of labour was generalised, but there were differences among different countries as for the drugs used and also in the use of controlled cord traction [18]. The second publication showed a high variability between the participating countries. In Spain, in particular, none of the evaluated units had a waiting time longer than 30 min. One limitation of this study is that it was only possible to evaluate variability between centres, as the data were obtained from the heads of maternity units, without taking into account the variability between individual professionals. Until now, the last study on the degree of implementation of active management in the third stage of labour in Spain was published in 2012, in which 1300 medical histories from 105 hospitals were reviewed, giving a result of 21.4% of implementation, well below the figures in our study [28]. Both studies’ results are not comparable as, in that study, medical histories were reviewed, and in our study, the professionals were asked directly.

According to several CPGs [7,24] and the WHO [8], the preferred drug for active management is oxytocin, which is in line with our study in which it was used by 71.6% of professionals. However, a Cochrane meta-analysis published in 2018 concluded that a combination of ergometrine plus oxytocin or a combination of carbetocin and misoprostol plus oxytocin are more effective uterotonics than oxytocin alone [15]. Furthermore, there is currently no consensus on the most appropriate dose or route of administration. The most usual recommendation is to administer 5 or 10 International Units (IU) intravenous (IV) or intramuscular (IM) oxytocin [29], with the route of administration having no bearing on the extent of blood loss prevention [30], so the decision is made by the professional attending the birth [29].

In this regard, in our study we observe that 55.7% of professionals used a dose of 10 IU and 35.0% used 5 IU, with the most common route of administration being IV bolus.

Among the factors associated with the least probability of using uterotonics during the third stage of labour we find the time elapsed since the completion of the training. Professionals that completed their training after 2007 and those still in training were more likely to use uterotonics than those who had completed their training before 2007. In the same line, other authors have identified that the longer the time elapsed since qualifying, the more difficulties there are in applying evidence-based clinical practices [31].

Another factor related to an increased use of uterotonics is the size of the hospital. The probability of uterotonics being used was especially high (>90%) in hospitals with more than 4000 births per year. In this case, bigger hospitals tend to have better quality indicators, [32] which is probably due to a higher degree of protocolisation of procedures.

The third identified factor was that professionals attended home births independently of whether they also worked in a hospital setting. These professionals were less likely to use uterotonics (32.7%) than those that did not attend home births (77.0%), probably with the aim of limiting interventions during the birth [33,34,35,36,37]. For this reason, it was decided to conduct a sub-analysis comparing professionals who attended home births and those who did not. It was proven that the former group used a more expectant approach, with lower doses and longer waiting times before performing manual delivery. With regard to the professional attending the birth, differences were found between midwives and gynaecologists. The first were less likely to use uterotonics during the third stage of labour and the waiting time before considering manual delivery was longer in their case, coinciding with the results of two US studies [19,20].

### 4.3. Strengths and Limitations

One limitation of the study is the possibility of a selection bias in the design of the study due to the fact that more midwives than gynaecologists participated. However, this reflects the actual practice in Spain, as eutocic births are habitually attended by midwives. In this sense, a complementary analysis was performed by using the profession variable as a weighting factor, observing no relevant differences as compared to the non-weighted analysis. Another severe study limitation is the low response rate of about 6.4%, since it carries an unknown risk of bias. One of the biggest strengths of the study is that it is the first study conducted in Spain to find out how this phase of labour is managed with a large sample which reveals the variability among professionals. Furthermore, the results of this study can serve as a basis for new research in this field to establish comparisons and healthcare policies aimed at improving training, and strengthening knowledge of and adherence to evidence-based clinical practices that have already been successful in other hospitals [38].

## 5. Conclusions

In Spain, there is a significant clinical variability among obstetrics professionals with regard to the management of the third stage of labour in normal births. Part of this variability can be attributed to professional and work environment factors. More research is needed to determine the most appropriate procedures for this stage of labour, which can then serve as the basis for professionals to draw up consensus statements and reduce variability in clinical practice.

## Figures and Tables

**Table 1:** Professional role and work environment.

Variable	*n* (%)	Response Rate of the Whole Population *n* (%)
Age		
≤25 years	113 (10.7)	
26–30 years	271 (25.7)	
31–35 years	188 (17.8)	
36–40 years	163 (15.5)	
41–45 years	134 (12.7)	
46–50 years	76 (7.2)	
51–55 years	51 (4.8)	
>55 years	58 (5.5)	
Gender		
Male	114 (10.8)	
Female	940 (89.2)	
Profession		
Midwife	797 (75.6)	797/9013 × 100 = 8.8%
Trainee midwife	97 (9.2)	97/989 × 100 = 9.8%
Gynaecologist	116 (11.0)	116/5616 × 100 = 2.1%
Trainee gynaecologist	44 (4.2)	44/1732 × 100 = 2.5%
Year of completion of training		
Before 2007	281 (26.7)	
After 2007	631 (59.9)	
In training	142 (13.5)	
Works in a public healthcare centre		
No	37 (3.5)	
Yes	1017 (96.5)	
Works in a private healthcare centre		
No	904 (85.8)	
Yes	150 (14.2)	
Attends home births		
No	1011 (95.9)	
Yes	43 (4.1)	
Works in Primary Care		
No	853 (80.9)	
Yes	201 (19.1)	
Number of births per year at the hospital they work at		
<500 births	91 (8.6)	
500–1000 births	165 (15.7)	
1000–2000 births	343 (32.5)	
2000–4000 births	283 (26.9)	
>4000 births	172 (16.3)	
Trainees at the hospital they work at		
No trainees	196 (18.6)	
Trainee midwives only	47 (4.5)	
Trainee gynaecologists only	56 (5.3)	
Both specialities	755 (71.6)	

**Table 2:** Clinical practices during the third stage of labour for all professionals.

Questions	*n* (%)	*n* (%) Weighted
Existence of a protocol for the management of the third stage of labour at the hospital		
No	292 (27.7)	272 (25.8)
Yes, but each professional applies his/her own criteria	223 (21.2)	209 (19.8)
Yes, and the majority of professionals apply it	539 (51.1)	573 (54.4)
Management of the third stage of labour in vaginal births		
Expectant or physiological	180 (17.1)	162 (15.4)
Active management with controlled cord traction only	80 (7.6)	96 (9.1)
Active management with use of uterotonics only	267 (25.3)	265 (25.1)
Active management with both controlled cord traction and use of uterotonics	527 (50.0)	531 (50.4)
Drug administered in the immediate postpartum period with physiological bleeding		
None	264 (25.0)	249 (23.6)
Oxytocin	755 (71.6)	760 (72.1)
Carbetocin	10 (0.9)	12 (1.1)
Methylergometrine maleate (Methergine®)	9 (0.9)	9 (0.8)
Misoprostol (Cytotec®)	12 (1.2)	18 (1.8)
Other	4 (0.4)	7 (0.6)
Frequency of administration of oxytocin in perfusion in the immediate postpartum period		
Never	67 (6.4)	59 (5.6)
Rarely	170 (16.1)	161 (15.2)
Occasionally	197 (18.7)	219 (20.7)
Frequently	203 (19.3)	207 (19.6)
Always	417 (39.6)	408 (38.7)
Situations in which oxytocin is administered during the immediate postpartum period in vaginal births with physiological bleeding		
Never	78 (7.4)	79 (7.5)
Only if clinically indicated	95 (9.0)	94 (8.9)
In women with risks factors for bleeding	264 (25.0)	258 (24.5)
Systematically	617 (58.5)	624 (59.2)
Oxytocin dose administered in the immediate postpartum period in vaginal births with physiological bleeding		
10 IU oxytocin	127 (12.0)	122 (11.5)
20 IU oxytocin	371 (35.2)	391 (37.0)
30 IU oxytocin	335 (31.8)	344 (32.6)
Variable dose depending on a protocolised checklist of risk factors for bleeding	69 (6.5)	55 (5.2)
Variable dose depending on risk factors according to my own criteria	152 (14.4)	143 (13.6)
Waiting time in a physiological delivery before considering it necessary for a gynaecologist to assess the need for manual removal of the placenta		
20 min	32 (3.0)	47 (4.5)
30 min	556 (52.8)	636 (60.3)
40 min	106 (10.1)	92 (8.7)
50 min	37 (3.5)	34 (3.2)
60 min	273 (25.9)	207 (19.7)
More than 60 min	50 (4.7)	38 (3.6)

IU: International Units.

**Table 3:** Clinical practices during the third stage of labour, only for professionals that used uterotonics in the third stage of labour (*n* = 794).

Questions	*n* (%)	*n* (%) Weighted
Situations in which uterotonics are administered in the third stage of labour	(*n* = 769)	(*n* = 769)
Only under doctor’s orders	2 (0.3)	1 (0.2)
Only in women with risk factors evaluated according to a protocolised checklist system	1 (0.1)	1 (0.1)
Only in women with risk factors that I think are relevant	36 (4.7)	27 (3.6)
Habitually	173 (22.5)	178 (23.2)
Always, unless there is some preventing reason	557 (72.4)	560 (73.0)
Missing values	25 (3.1)	28 (3.6)
Reason for not administering uterotonics in the third stage of labour: Nobody available to administer the drug	(*n* = 755)	(*n* = 755)
No	347 (46.0)	347 (46.2)
Yes	408 (54.0)	405 (53.8)
Missing values	39 (4.9)	44 (5.6)
Reason for not administering uterotonics in the third stage of labour: Only when I forget or due to lack of preparation	(*n* = 754)	(*n* = 754)
No	510 (67.6)	489 (64.9)
Yes	244 (32.4)	264 (35.1)
Missing values	40 (5.0)	43 (5.4)
Reason for not administering uterotonics in the third stage of labour: Lack of preparation in earlier-than-expected births	(*n* = 750)	(*n* = 750)
No	245 (32.7)	246 (33.0)
Yes	505 (67.3)	500 (67.0)
Missing values	44 (5.5)	50 (6.3)
Reason for not administering uterotonics in the third stage of labour: To donate cord blood	(*n* = 751)	(*n* = 751)
No	374 (49.8)	364 (48.5)
Yes	377 (50.2)	387 (51.5)
Missing values	43 (5.4)	46 (5.7)
Reason for not administering uterotonics in the third stage of labour: When the mother has expressed a desire for physiological delivery	(*n* = 749)	(*n* = 749)
No	409 (54.6)	446 (59.8)
Yes	340 (45.4)	300 (40.2)
Missing values	45 (5.7)	50 (6.2)
Drug and dose used for delivery with uterotonics	(*n* = 766)	(*n* = 766)
Oxytocin 3 IU	11 (1.5)	8 (1.0)
Oxytocin 5 IU	268 (35.0)	260 (34.0)
Oxytocin 10 IU	427 (55.7)	430 (56.2)
Oxytocin 5 or 10 IU (variable dose)	47 (6.1)	50 (6.5)
Methylergometrine maleate (Methergine®)	2 (0.3)	1 (0.2)
Syntometrine	1 (0.1)	1 (0.1)
Other	10 (1.3)	15 (2.0)
Missing values	28 (3.5)	30 (3.8)
Time of administration of the uterotonic drug in the third stage of labour	(*n* = 767)	(*n* = 767)
When the anterior shoulder emerges	455 (59.3)	505 (65.7)
When the baby is born	233 (30.4)	185 (24.0)
When the umbilical cord is clamped	60 (7.8)	60 (7.8)
When the placenta is expelled	15 (2.0)	16 (2.1)
No criteria	4 (0.5)	3 (0.3)
Missing values	27 (3.4)	27 (3.4)
Route of administration of the uterotonic drug in the third stage of labour	(*n* = 766)	(*n* = 766)
Intramuscular	77 (10.1)	76 (10.0)
Intravenous bolus	587 (76.6)	588 (76.8)
Continuous intravenous infusion	102 (13.3)	101 (13.2)
Missing values	28 (3.5)	30 (3.8)
Use of controlled cord traction	(*n* = 758)	(*n* = 758)
I do not use cord traction	121(15.96)	121(15.96)
I only use cord traction	39 (5.15)	39 (5.15)
Credé manoeuvre	187 (24.67)	187 (24.67)
Brandt–Andrews manoeuvre	411 (54.22)	411 (54.22)
Missing values	36 (4.5)	36 (4.5)
Waiting time in the third stage of labour with administration of uterotonics before considering it necessary to ask a gynaecologist to assess the need for manual removal of the placenta	(*n* = 764)	(*n* = 764)
10 min	9 (1.2)	12 (1.6)
20 min	47 (6.2)	76 (10.0)
30 min	644 (84.3)	621 (81.5)
40 min	43 (5.6)	38 (4.9)
50 min	5 (0.7)	3 (0.4)
More than 50 min	16 (2.1)	11 (1.5)
Missing values	30 (3.8)	34 (4.3)

IU: International Units.

**Table 4:** Factors related to the administration of a uterotonic drug during the third stage of labour.

Variable	Administration of a Uterotonic Drug			
	No (*N* = 260) *n* (%)	Yes (*N* = 794) *n* (%)	OR CI 95%	*ORa CI 95%
Age				
≤25 years	19 (16.8)	94 (83.2)	1 (ref.)	
26–30 years	57 (21.0)	214 (79.0)	0.75 (0.42–1.34)	
31–35 years	46 (24.5)	142 (75.5)	0.62 (0.34–1.13)	
36–40 years	39 (23.9)	124 (76.1)	0.64 (0.34–1.18)	
41–45 years	35 (26.1)	99 (73.9)	0.57 (0.30–1.06)	
46–50 years	27 (35.5)	49 (64.5)	0.36 (0.18–0.72)	
51–55 years	20 (39.2)	31 (60.8)	0.31 (0.14–0.66)	
>55 years	17 (29.3)	41 (70.7)	0.48 (0.23–1.03)	
Gender				
Male	36 (31.6)	78 (68.4)	1 (ref.)	
Female	224 (23.8)	716 (76.2)	1.47 (0.96–2.25)	
Profession				
Midwife	211 (26.5)	586 (73.5)	1 (ref.)	
Trainee midwife	14 (14.4)	83 (85.6)	2.13 (1.18–3.84)	
Gynaecologist	28 (24.1)	88 (75.9)	1.13 (0.71–1.78)	
Trainee gynaecologist	7 (15.9)	37 (84.1)	1.90 (0.83–4.33)	
Completion of training				
Before 2007	96 (34.2)	185 (65.8)	1 (ref.)	1 (ref.)
After 2007	142 (22.5)	489 (77.5)	1.78 (1.31–2.49)	1.57 (1.13–2.18)
Currently in training	22 (15.5)	120 (84.5)	2.83 (1.68–4.74)	1.94 (1.13–3.34)
Works in a public healthcare centre				
No	16 (43.2)	21 (56.8)	1 (ref.)	
Yes	244 (24.0)	773 (76.0)	2.41 (1.24-4.69)	
Works in a private healthcare centre				
No	49 (32.7)	101 (67.3)	1 (ref.)	
Yes	211 (23.3)	693 (76.7)	0.62 (0.43-0.91)	
Attends home births				
No	233 (23.0)	778 (77.0)	1 (ref.)	1 (ref.)
Yes	27 (62.8)	16 (37.2)	0.17 (0.09–0.33)	0.23 (0.12–0.47)
Works in primary care				
No	213 (25.0)	640 (75.0)	1 (ref.)	
Yes	47 (23.4)	154 (76.6)	1.09 (0.76–1.56)	
Number of births per year in their hospital				
<500 births	48 (52.7)	43 (47.3)	1 (ref.)	1 (ref.)
500–1000 births	49 (29.7)	116 (70.3)	2.64 (1.55–4.49)	2.12 (1.22–3.67)
1000–2000 births	104 (30.3)	239 (69.7)	2.56 (1.60–4.11)	1.98 (1.20–3.24)
2000–4000 births	43 (15.2)	240 (84.8)	6.23 (3.68–10.52)	4.89 (2.84–8.43)
>4000 births	16 (9.3)	156 (90.7)	10.88 (5.63–21.03)	7.95 (4.02–15.72)
Professionals in training at the hospital				
No professionals in training	89 (45.4)	107 (54.6)	1 (ref.)	
Trainee midwives only	15 (31.9)	32 (68.1)	1.77 (0.90–3.48)	
Trainee gynaecologists only	13 (23.2)	43 (76.8)	2.75 (1.39–5.43)	
Both specialities	143 (18.9)	612 (81.1)	3.56 (2.54–4.97)	

## Appendix A

**Table A1:** Attendance at home births and practices in the immediate postpartum period.

Questions on Practices in the Immediate Postpartum Period	Attends Home Births		
	No (*N* = 1011) *n* (%)	Yes (*N* = 43) *n* (%)	*P*-value
Drug administered in the immediate postpartum period with physiological bleeding			0.002 *
None	242 (23.9)	22 (51.2)	
Oxytocin	735 (72.7)	20 (46.5)	
Carbetocin	10 (1.0)	0 (0.0)	
Methylergometrine maleate (Methergine®)	9 (0.9)	0 (0.0)	
Misoprostol (Cytotec®)	15 (1.5)	1 (2.3)	
Frequency of administration of oxytocin in perfusion in the immediate postpartum period			<0.001 **
Never	55 (5.4)	12 (27.9)	
Rarely	162 (16.0)	8 (18.6)	
Occasionally	188 (18.6)	9 (20.9)	
Frequently	194 (19.2)	9 (20.9)	
Always	412 (40.8)	5 (11.6)	
Situations in which oxytocin is administered during the immediate postpartum period in vaginal births with physiological bleeding			<0.001*
Never	62 (6.1)	16 (37.2)	
Only if medically indicated	91 (9.0)	4 (9.3)	
In women with risk factors for bleeding	251 (24.8)	13 (30.2)	
Systematically	607 (60.0)	10 (23.3)	
Oxytocin dose administered in the immediate postpartum period in vaginal births with physiological bleeding			<0.001 **
10 IU oxytocin	107 (10.6)	20 (46.5)	
20 IU oxytocin	364 (36.0)	7 (16.3)	
30 IU oxytocin	327 (32.3)	8 (18.6)	
Variable dose depending on a protocolised checklist of risk factors for bleeding	66 (6.5)	3 (7.0)	
Variable dose depending on risk factors according to my criteria	147 (14.5)	5 (11.6)	
Waiting time after administering uterotonic drugs in the third stage of labour before considering it necessary for a gynaecologist to assess the need for manual removal of the placenta			<0.001 **
10 min	12 (1.5)	0 (0.0)	
20 min	53 (6.4)	0 (0.0)	
30 min	685 (83.0)	13 (56.5)	
40 min	50 (6.1)	3 (13.0)	
50 min	6 (0.7)	2 (8.7)	
More than 50 min	19 (2.3)	5 (21.7)	
Waiting time in a physiological delivery before considering it necessary for a gynaecologist to assess the need for manual removal of the placenta			<0.001 **
20 min	32 (3.2)	0 (0.0)	
30 min	550 (54.4)	6 (14.0)	
40 min	97 (9.6)	9 (20.9)	
50 min	34 (3.4)	3 (7.0)	
60 min	261 (25.8)	12 (27.9)	
More than 60 min	37 (3.7)	13 (30.2)	

*: Pearson’s chi-squared test; **: Mann-Whitney U test.

## Appendix B

**Table A2:** Type of professional and practices in the immediate postpartum period.

Questions on Practices in the Immediate Postpartum Period	Professional Attending the Birth		
	Midwives (*N* = 894) *n* (%)	Gynaecologists (*N* = 160) *n* (%)	*P*-value
Drug administered in the immediate postpartum period with physiological bleeding			0.008 *
None	234 (26.2)	30 (18.8)	
Oxytocin	635 (71.0)	120 (75.0)	
Carbetocin	8 (0.9)	2 (1.3)	
Methylergometrine maleate (Methergine®)	8 (0.9)	1 (0.6)	
Misoprostol (Cytotec®)	9 (1.0)	7 (4.4)	
Frequency of administration of oxytocin in perfusion in the immediate postpartum period			0.667 **
Never	61 (6.8)	6 (3.8)	
Rarely	151 (16.9)	19 (11.9)	
Occasionally	156 (17.4)	41 (25.6)	
Frequently	169 (18.9)	34 (21.3)	
Always	357 (39.9)	60 (37.5)	
Situations in which oxytocin is administered during the immediate postpartum period in vaginal births with physiological bleeding			0.584 *
Never	67 (7.5)	11 (6.9)	
Only if medically indicated	77 (8.6)	18 (11.3)	
In women with risk factors for bleeding	229 (25.6)	35 (21.9)	
Systematically	521 (58.3)	96 (60.0)	
Oxytocin dose administered in the immediate postpartum period in vaginal births with physiological bleeding			0.238 *
10 IU oxytocin	110 (12.3)	17 (10.6)	
20 IU oxytocin	306 (34.2)	65 (40.6)	
30 IU oxytocin	283 (31.7)	52 (32.5)	
Variable dose depending on a protocolised checklist of risk factors for bleeding	64 (7.2)	5 (3.1)	
Variable dose depending on risk factors according to my criteria	131 (14.7)	21 (13.1)	
Waiting time after administering uterotonic drugs in the third stage of labour before considering it necessary for a gynaecologist to assess the need for manual removal of the placenta			<0.001 **
10 min	7 (1.0)	5 (3.7)	
20 min	28 (3.9)	25 (18.4)	
30 min	599 (84.1)	99 (72.8)	
40 min	47 (6.6)	6 (4.4)	
50 min	8 (1.1)	0 (0.0)	
More than 50 min	23 (3.2)	1 (0.7)	
Waiting time in a physiological delivery before considering it necessary for a gynaecologist to assess the need for manual removal of the placenta			<0.001 **
20 min	19 (2.1)	13 (8.1)	
30 min	431 (48.2)	125 (78.1)	
40 min	98 (11.0)	8 (5.0)	
50 min	34 (3.8)	3 (1.9)	
60 min	263 (29.4)	10 (6.3)	
More than 60 min	49 (5.5)	1 (0.6)	

*: Pearson’s chi-squared test; **: Mann–Whitney U test.
